# 伴有骨髓侵犯的滤泡性淋巴瘤临床特征与预后分析

**DOI:** 10.3760/cma.j.cn121090-20240613-00222

**Published:** 2024-12

**Authors:** 瑞 吕, 文婕 熊, 婷玉 王, 禹廷 阎, 齐 王, 颖 于, 薇 刘, 文阳 黄, 刚 安, 燕 徐, 德慧 邹, 录贵 邱, 树华 易

**Affiliations:** 1 中国医学科学院血液病医院（中国医学科学院血液学研究所），血液与健康全国重点实验室，国家血液系统疾病临床医学研究中心，细胞生态海河实验室，天津 300020 State Key Laboratory of Experimental Hematology, National Clinical Research Center for Blood Diseases, Haihe Laboratory of Cell Ecosystem, Institute of Hematology & Blood Diseases Hospital, Chinese Academy of Medical Science & Peking Union Medical College, Tianjin 300020, China; 2 天津医学健康研究院，天津 301600 Tianjin Institutes of Health Science, Tianjin 301600, China

**Keywords:** 淋巴瘤，滤泡性, 骨髓侵犯, 染色体核型, FLIPI评分, R-CHOP方案, Lymphoma, follicular, Bone marrow invasive, Karyotype, FLIPI score, R-CHOP therapy

## Abstract

**目的:**

总结伴有骨髓侵犯的滤泡性淋巴瘤（FL）患者临床特征及预后，并对治疗模式进行探讨。

**方法:**

纳入2013年1月至2022年12月中国医学科学院血液病医院连续收治的183例伴有骨髓侵犯且接受正规治疗的FL患者。回顾性收集并分析患者临床资料，采用Kaplan-Meier法及Cox回归模型进行生存预后的单因素及多因素分析。

**结果:**

中位年龄48（19～78）岁，男女比例0.9∶1。所有患者均有骨髓侵犯，LDH升高比例为27.8％，淋巴细胞计数>5×10^9^/L比例为42.1％，染色体异常及淋巴组织Ki-67指数≥30％比例分别为18.4％及48.6％；不同亚组比较：强化治疗组的淋巴细胞计数>5×10^9^/L、受累淋巴结数目≥5个以及发生骨髓染色体异常比例，均较R-CHOP（利妥昔单抗+环磷酰胺+阿霉素+长春地辛+泼尼松）组及核苷类似物（包括CD20单抗联合氟达拉滨及苯达莫司汀）组更高（均*P*<0.05）。R-CHOP组完全缓解率为39.1％，较强化治疗组（55.1％）及核苷类似物组（62.5％）低（*P*＝0.042）。生存分析：滤泡性淋巴瘤国际预后指数（FLIPI）高危［*HR*=1.910（95％ *CI* 1.036～3.522），*P*=0.038］、染色体异常核型［*HR*＝2.666（95％ *CI* 1.333～5.331），*P*＝0.006］以及R-CHOP方案治疗［*HR*＝2.287（95％ *CI* 1.140～4.591），*P*＝0.020］被证实为影响无进展生存（PFS）的独立不良预后因素；而24个月内疾病进展（POD24）为影响总生存的唯一独立不良预后因素［*HR*＝9.581（95％ *CI* 3.000～30.593），*P*<0.001］。

**结论:**

伴有骨髓侵犯的FL患者容易合并淋巴细胞计数增高、染色体异常及淋巴组织Ki-67指数增高等临床特征，FLIPI评分、染色体异常核型以及不同治疗方案均为影响PFS的不良预后因素。对于这部分患者，R-CHOP方案疗效欠佳。

## Abstract (en)

**Objective:**

This study aimed to summarize the clinical characteristics and prognosis of patients with bone marrow invasive follicular lymphoma（FL）and discuss the treatment modalities.

**Methods:**

This study included 183 consecutive patients with FL accompanied by bone marrow invasion and receiving regular treatment at the Hospital of Hematology, Chinese Academy of Medical Sciences, from January 2013 to December 2022. Clinical data were retrospectively collected and analyzed, and single and multifactorial analyses of survival prognosis were conducted with the Kaplan-Meier method and Cox regression model.

**Results:**

The median age was 48（range: 19–78）years, and the male-to-female ratio was 0.9∶1. All of the patients had bone marrow invasion, 27.8％ had increased lactate dehydrogenase levels, 42.1％ had lymphocyte counts of >5×10^9^/L, 18.4％ had abnormal chromosomal karyotypes, and 48.6％ had Ki-67 index of ≥30％ in lymphoid tissue. Comparison of different subgroups: lymphocyte counts of >5×10^9^/L, number of lymph nodes of ≥5 involved, and proportion of bone marrow chromosomal abnormalities occurring were higher in the anthracycline-intensive treatment group than in the rituximab, cyclophosphamide, doxorubicin, vincristine, and prednisone（R-CHOP）protocol and the nucleoside analog（including CD20 monoclonal antibody in combination with fludarabine and bendamustine）groups（all *P*<0.05）. The complete remission rate was 39.1％ in the conventional R-CHOP group, which was lower and statistically significant than that in the intensive treatment group（55.1％）and the nucleoside analog group（62.5％）（*P*＝0.042）. The multivariate analysis for survival analysis revealed high risk of FLIPI（*HR*＝ 1.910, 95％ *CI* 1.036–3.522, *P*＝0.036）, chromosomal abnormalities karyotype（*HR*＝2.666，95％ *CI* 1.333–5.331, *P*＝0.006）, and conventional R-CHOP treatment（*HR*＝2.287, 95％ *CI* 1.140–4.591, *P*＝0.020）were the independent adverse prognostic factors affecting progression-free survival（PFS）, whereas POD24 was the only independent adverse prognostic factor affecting overall survival（OS）adverse prognostic factor（*HR*＝9.581, 95％ *CI* 3.000–30.593, *P*<0.001）.

**Conclusion:**

The clinical presentations of patients with bone marrow invasive FL were easy to combine the clinical features, including increased lymphocyte count, chromosomal abnormalities, and Ki-67 index in lymphoid tissues. The FLIPI score, chromosomal abnormal karyotype, and high-lymphoid-tissue Ki-67 index were the poor prognostic factors influencing PFS. R-CHOP therapy demonstrated a poor prognosis in this group of patients.

滤泡性淋巴瘤（FL）为常见的惰性淋巴瘤，绝大多数患者伴有骨髓侵犯。既往欧洲肿瘤内科学会（ESMO）指南[1]及美国国立癌症综合网络（NCCN）指南[2]中均推荐骨髓穿刺及活检作为FL诊断及治疗过程中的常规检测手段，但近期有学者对骨髓检测的价值提出一定质疑[3]，同时对于骨髓侵犯患者的临床特征及治疗，国内外均缺乏相应总结。因此，本研究通过分析伴有骨髓侵犯的FL患者的临床资料，拟对其临床特征及预后加以总结，并对其治疗模式进行初步探讨。

## 病例和方法

1. 病例：纳入2013年1月至2022年12月中国医学科学院血液病医院收治入院的183例伴有骨髓侵犯且达到治疗指征，并接受规范治疗的FL患者，所有患者在诊断时均通过病理及PET-CT等临床检查结果排除了转化，且同时通过流式细胞术及病理检验证实存在骨髓侵犯。收集患者临床资料，包括血液生化、影像、病理及遗传学等检查结果。对所有纳入的患者进行总体临床特征分析及遗传学特点总结。针对接受不同治疗方案的患者进行基线比较及疗效预后分析，并针对随访时间>2年患者计算其24个月内疾病进展（POD24）发生率。本研究经中国医学科学院血液病医院伦理委员会批准（批件号：IIT2021030-EC-1），所有患者均已签署知情同意书。

2. 诊断及分期标准：诊断标准均参照世界卫生组织（WHO）2022年淋巴组织肿瘤分类标准[4]；其中，未行淋巴结切检，而仅依靠骨髓诊断的患者需具备典型的免疫表型，同时病理表现满足FL侵犯骨髓的典型特征及免疫组化改变，且绝大多数具备IgH/BCL2易位，并完全排除转化。分期依据改良Ann Arbor分期标准，因纳入患者均存在骨髓侵犯，故均为Ⅳ期。染色体核型采用G显带的方法，同时加用DSP30联合IL-2增加刺激剂培养以提高检出率。采用间期FISH并分别应用BCL2/IgH融合探针，TP53、ATM及CDKN2A缺失信号探针，CEP12数目探针等来检测常见异常染色体片段。此外，分子遗传学方面，对部分患者还应用靶向测序方法针对骨髓及肿瘤组织的常见位点突变进行检测。

3. 治疗方案及疗效标准：纳入的183例患者中，有110例患者接受R-CHOP（利妥昔单抗+环磷酰胺+阿霉素+长春地辛+泼尼松）方案化疗（R-CHOP组）；24例患者接受以核苷类似物为基础的方案化疗（核苷类似物组），其中RFC（利妥昔单抗+氟达拉滨+环磷酰胺）方案14例、BR（苯达莫司汀+利妥昔单抗）方案10例；强化治疗方案49例（强化治疗组），其中R-HyperCVAD（利妥昔单抗+大剂量环磷酰胺+长春地辛+阿霉素+地塞米松）方案7例、REDOCH（利妥昔单抗+依托泊苷+地塞米松+长春新碱+环磷酰胺+阿霉素）方案18例、R2-CHOP（利妥昔单抗+来那度胺+环磷酰胺+阿霉素+长春地辛+泼尼松）方案24例。所有患者疗效评价标准均参照2014年Lugano国际工作组评价标准[5]，包括完全缓解（CR）、部分缓解（PR）、疾病稳定（SD）、疾病进展（PD）、总有效率（ORR）为CR率与PR率之和。该研究基于国家血液系统疾病淋巴瘤队列（NICHE），同时符合《赫尔辛基宣言》。

4. 随访：通过查阅病例及电话进行随访，所有患者随访时间截至2024年6月。其中29例患者因失访无法评估预后，14例患者因随访不足24个月无法计算POD24发生率。总生存（OS）期定义为自本次疾病明确诊断至因任何原因死亡或末次随访时间。无进展生存（PFS）期定义为自本次疾病明确诊断至疾病进展或末次随访时间。将自诊断并正规治疗后发生24个月内疾病进展定义为POD24[6]。

5. 统计学处理：应用SPSS 26.0软件进行统计学分析。计数资料用例数（百分比）表示，计量资料用*M*（范围）或*x*±*s*表示。采用*χ*^2^检验、Fisher精确概率法、Kruskal-Wallis检验进行组间比较，采用Kaplan-Meier法及Cox回归模型进行生存预后的单因素及多因素分析。双侧*P*<0.05为差异有统计学意义。

## 结果

1. 一般资料：如表1所示，患者中位年龄48（19～78）岁，男87例，女96例，血红蛋白减低及血小板减低的比例分别为24.6％及10.9％，LDH增高的比例为27.8％，淋巴细胞计数>5×10^9^/L比例为42.1％，染色体异常及淋巴组织Ki-67指数≥30％比例分别为18.4％及48.6％。在R-CHOP组、核苷类似物组及强化治疗组中，通过比较其基线特征，可见淋巴细胞计数>5×10^9^/L、受累淋巴结数目≥5个以及发生骨髓染色体异常的比例，均以强化治疗组比例更高（均*P*<0.05），腹腔肿块长径>6 cm的比例则以核苷类似物组相对偏低（*P*＝0.018）。

**表1:** 不同治疗组的滤泡性淋巴瘤患者基线临床特征

临床特征	R-CHOP组（110例）	强化治疗组（49例）	核苷类似物组（24例）	统计量	*P*值
年龄［岁，*M*（范围）］	47（20~78）	45（19~66）	50（25~69）	2.133	0.121
男性［例（％）］	47（42.7）	29（59.2）	11（45.8）	3.713	0.156
淋巴细胞计数［×10^9^/L，*M*（范围）］	2.08（0.27~73.56）	9.98（0.31~116.30）	1.80（0.94~31.92）	4.614	0.011
淋巴细胞计数>5×10^9^/L［例（％）］	39（35.5）	30（61.2）	8（33.3）	10.103	0.006
HGB<120 g/L［例（％）］	29（26.4）	11（22.4）	5（20.8）	0.490	0.783
PLT<100×10^9^/L［例（％）］	14（12.7）	5（10.2）	1（4.2）	1.519	0.468
LDH>247 U/L［例（％）］	27（24.5）	15（30.6）	9（37.5）	1.895	0.388
受累淋巴结数目≥5个［例（％）］	71（64.5）	45（91.8）	19（79.2）	13.464	0.001
巨脾［例（％）］	13（11.8）	11（22.4）	3（12.5）	3.158	0.206
腹腔肿块长径>6 cm［例（％）］	41（37.2）	19（38.8）	2（8.3）	8.081	0.018
结外受累>1处［例（％）］	44（40.0）	26（53.1）	15（62.5）	4.960	0.084
FLIPI高危组［例（％）］	35（31.8）	21（42.9）	9（37.5）	1.851	0.396
病理3a级［阳性例数/检测例数（％）］	17/83（20.5）	2/20（10.0）	10/39（25.6）	1.991	0.370
Ki-67指数≥30％［阳性例数/检测例数（％）］	32/62（51.6）	15/33（45.5）	5/10（50.0）	0.251	0.882
染色体异常［阳性例数/检测例数（％）］	18/100（18.0）	11/46（23.9）	0/22（0）	6.053	0.048
细胞遗传学异常［阳性例数/检测例数（％）］	5/94（5.3）	2/46（4.3）	1/18（5.5）	0.071	0.965

**注** R-CHOP：利妥昔单抗+环磷酰胺+阿霉素+长春地辛+泼尼松；FLIPI：滤泡性淋巴瘤国际预后指数

2. 遗传学特征：有5％（8/158）的患者存在细胞遗传学异常，其中包括TP53缺失4例、MYC缺失1例、CEP12异常2例及CDKN2A缺失1例；17.3％（29/168）患者染色体为异常核型。分子遗传学方面，66例患者发生基因突变，突变发生比例前5位的基因分别为KMT2D（81.8％）、CREBBP（68.2％）、BCL2（31.8％）、EP300（22.7％）及BCL6（16.7％）。

3. 疗效情况：如表2所示，3个治疗组ORR为84.7％、CR率46.4％，RCHOP组、强化治疗组、核苷类似物组ORR分别为81.8％、87.8％、91.6％（*P*＝0.376）；CR率分别为39.1％、55.1％、62.5％（*P*＝0.042）。

**表2:** 不同治疗组滤泡性淋巴瘤患者疗效及3～4级血液学不良反应情况［例（％）］

疗效及不良反应	R-CHOP组（110例）	强化治疗组（49例）	核苷类似物组（24例）	统计量	*P*值
CR	43（39.1）	27（55.1）	15（62.5）	6.355	0.042
PR	47（42.7）	16（32.7）	7（29.2）	2.422	0.298
SD	18（16.4）	5（10.2）	2（8.3）	1.755	0.416
PD	2（1.8）	1（2.0）	0（0）	0.471	0.790
3～4级白细胞减少	36（32.7）	25（51.0）	7（29.2）	5.614	0.060
3～4级粒细胞减少	20（18.2）	24（48.9）	7（29.2）	16.018	<0.001
3～4级淋巴细胞减少	22（20.0）	11（22.4）	14（58.3）	15.534	<0.001
3～4级血红蛋白减少	6（5.4）	5（10.2）	1（4.2）	1.506	0.471
3～4级血小板减少	4（3.6）	14（28.6）	0（0）	26.780	<0.001

**注** R-CHOP：利妥昔单抗+环磷酰胺+阿霉素+长春地辛+泼尼松；CR：完全缓解；PR：部分缓解；SD：疾病稳定；PD：疾病进展

4. 不良反应：血液学不良反应：强化治疗组中，发生3～4级粒细胞减少及3～4级血小板减少的比例远高于R-CHOP组及核苷类似物组；而发生3～4级淋巴细胞减少的比例却在核苷类似物组最为明显（均*P*<0.001），如表2所示。非血液学不良反应：R-CHOP组3例（2.7％）患者发生感染，强化治疗组8例（16.3％）患者发生感染，核苷类似物组4例（16.6％）患者发生感染；2例（4.1％）患者出现皮疹，但均未影响总体治疗，无任何严重不良事件发生。

5. 随访及预后分析：中位随访59（53.3～64.6）个月，患者总体5年PFS率和OS率分别为（64.9±4.8）％、（86.8±3.3）％。发生POD24的患者25例（17.8％），但3个治疗组之间差异无统计学意义（21.2％对17.0％对10.5％，*P*＝0.269）。预后分析中，影响PFS的不良预后因素包括滤泡性淋巴瘤国际预后指数（FLIPI）高危、治疗方式、骨髓染色体异常核型及淋巴结Ki-67指数≥30％；影响OS的不良预后因素包括FLIPI高危及POD24（表3）。

**表3:** 滤泡性淋巴瘤患者的单因素及多因素预后分析

因素	无进展生存				总生存			
	单因素分析		多因素分析		单因素分析		多因素分析	
	*HR*（95％ *CI*）	*P*值	*HR*（95％ *CI*）	*P*值	*HR*（95％ *CI*）	*P*值	*HR*（95％ *CI*）	*P*值
FLIPI高危（是/否）	1.958（1.097~3.496）	0.023	1.910（1.036~3.522）	0.038	2.729（1.014~7.345）	0.047	1.698（0.603~4.783）	0.317
染色体异常核型（是/否）	2.656（1.339~5.268）	0.005	2.666（1.333~5.331）	0.006	2.627（0.895~7.714）	0.079	–	–
R-CHOP方案治疗（是/否）	2.267（1.171~4.388）	0.015	2.287（1.140~4.591）	0.020	1.032（0.895~7.714）	0.590	–	–
Ki-67指数≥30％（是/否）	2.231（1.005~4.952）	0.049	–	–	1.500（0.244~4.367）	0.598	–	–
POD24（是/否）	–	–	–	–	10.482（3.332~32.978）	<0.001	9.581（3.000~30.593）	<0.001

**注** FLIPI：滤泡性淋巴瘤国际预后指数；R-CHOP：利妥昔单抗+环磷酰胺+阿霉素+长春地辛+泼尼松；POD24：24个月内疾病进展；–：未计算

由于部分骨髓诊断的患者未能行淋巴结切检，导致数据Ki-67指数缺失较多且随访时间相对较短，且其单因素分析曲线存在明显交叉，不符合多因素分析的纳入原则，因此多因素分析并未将Ki-67指数纳入，结果示：FLIPI高危、染色体异常核型以及传统R-CHOP治疗为影响PFS的独立不良预后因素；POD24为影响OS的独立不良预后因素（表3）。

## 讨论

FL是起源于生发中心B细胞的惰性肿瘤，中位年龄60～65岁，通常以广泛淋巴结肿大、骨髓侵犯及脾大为主要临床特征，其骨髓侵犯比例占总体FL的50％～60％，但伴有骨髓侵犯的FL患者是否具备独特的临床特征并不清楚。据文献报道，在所有FL患者中，血红蛋白降低及血小板减低比例约10％，淋巴细胞计数增高比例5％～10％[7]–[8]。本研究中伴有骨髓侵犯的FL患者中位年龄<50岁，血红蛋白及血小板下降比例为10％～20％，淋巴细胞计数增高的比例超过40％，均较文献报道偏高，这提示伴有骨髓侵犯的FL患者更容易出现血象异常及淋巴细胞计数增高的临床特征。

骨髓侵犯在基于PRIMA临床试验的滤泡性淋巴瘤预后评分指数（PRIMA-PI）中为独立不良因素[9]，但针对该类特定患者的疗效及预后缺乏相应研究。本研究患者总体CR率为46.4％，5年PFS率为（64.9±4.8）％，R-CHOP组CR率为39.1％，5年PFS率仅为40％，对比FOLL005[10]及PRIMA[11]临床试验结果均更差（FL005及PRIMA试验中CR率为73％及51.2％，5年PFS率为74.9％及55.7％），其原因考虑一方面伴有骨髓侵犯的FL患者本身血象异常及相应染色体复杂核型比例高对预后的不良影响，另一方面，以淋巴细胞增高为表现的白血病样FL患者在队列中的比例超过40％，尽管预后仍有争议，但其PFS期及OS期均较对照组缩短[12]，也成为影响总体疗效的因素之一。在多因素分析方面，影响该组患者预后因素包括FLIPI评分、异常染色体核型、治疗方式及Ki-67指数，但因Ki-67指数生存曲线存在明显交叉未纳入多因素分析，该项结果对临床有一定借鉴意义：①FLIPI评分系统源于利妥昔单抗应用前时代的研究，但在利妥昔单抗时代亦具独立预后价值[7]，在本组伴有骨髓侵犯的FL患者中，其预后价值亦有验证，进一步证实临床中FLIPI评分在FL的重要性；②伴有骨髓侵犯的FL患者由于其治疗后骨髓形态及病理往往先于影像学转阴，部分学者提出可以减少骨髓穿刺以避免不必要的有创损伤[3]，但在本组患者中，染色体异常核型比例接近20％且明确与PFS相关，提示临床对于FL骨髓穿刺的价值并不能完全忽视，尤其需要注重基线期骨髓染色体核型的检测；③Ki-67指数代表着肿瘤增殖活性，即便在低级别FL中亦有约五分之一患者≥30％，且与PRIMA-PI联合能更好提示预后[13]，但由于受骨髓脱钙影响，在具体临床操作中有时容易忽略，但本研究的结果显示了其对PFS的不良影响，因此提示在伴有骨髓侵犯的FL中进行淋巴组织活检及其相应Ki-67指数的检测仍有必要。

此外，伴有骨髓侵犯的FL患者的最佳治疗方案亦不明确。在总体FL指南中，传统以R-CHOP方案为标准治疗，但随着stiL研究[14]及BRIGHT研究[15]的出现，欧美国家目前一线主流为BR方案。但是，对于不同高危FL，其最佳方案却不一定是BR方案。而对FL早期的预知及分层治疗，仍是研究的重点难点，也是国内外学者不断探索的方向：早期研究[16]认为FL3a患者可能从BR方案上获益更多，但近年的多中心回顾研究却得到不一样的结论[17]；基线PET-CT对治疗有一定指导作用，起病最大标准摄取值（SUVmax）>18选用R-CHOP方案比BR方案获益更大[18]，但是否为最佳方案仍不能明确；R2-CHOP方案治疗初诊FL的Ⅱ期临床试验，发现其有可能提高FLIPI高危患者的疗效[19]，但尚有待进一步验证推广；我们之前在高侵袭的年轻的高危FL患者中探索强化治疗模式，发现部分患者获得了长期生存，但也仅局限于个别患者，仍有待进一步研究[20]。本队列中，无论核苷类似物组，还是强化治疗组，均较R-CHOP组提示更高的CR率，且多因素分析中，对比R-CHOP组有独立预后意义（*P*＝0.020），且强化治疗组在淋巴细胞计数、受累淋巴结数目、发生骨髓染色体异常等临床特征方面，均较R-CHOP组患者更为高危。因此，对于骨髓侵犯患者的治疗，R-CHOP方案可能并不是最佳选择，强化治疗方法在基线更差的情况下，反而疗效提高，提示其具有治疗前景，但由于其伴随着更为明显的血液学不良反应，最佳治疗模式仍值得进一步探索。此外，Ki-67指数≥30％的患者尽管在R-CHOP方案组提示预后不良（*P*<0.01），但通过强化治疗，其不良预后一定程度被抵消了（*P*＝0.803），据此我们推测Ki-67高危的患者有可能通过强化治疗获得更好的疗效，但其结论亦有待进一步验证。

综上，伴有骨髓侵犯的FL患者，容易合并淋巴细胞计数增高、染色体异常及淋巴组织Ki-67指数增高等临床特征。尽管POD24仍为OS的独立预后因素，但FLIPI评分高危，染色体异常核型，淋巴组织Ki-67指数≥30％对PFS的预后价值充分提示，在常规FLIPI评分基础上，对该部分患者同时完善基线期骨髓染色体检测及淋巴组织病理Ki-67指数的重要性。在治疗模式上，伴有骨髓侵犯的FL患者很难从R-CHOP方案上获益，BR方案、强化治疗方案均能在R-CHOP方案基础上提高疗效，但其最佳治疗模式有待进一步探索。由于本研究样本数有限且为回顾性研究，随访时间较短，其结论有待更大样本的研究以及随机对照临床试验进一步验证。

## References

[1] Dreyling M, Ghielmini M, Rule S (2021). Newly diagnosed and relapsed follicular lymphoma: ESMO Clinical Practice Guidelines for diagnosis, treatment and follow-up[J]. Ann Oncol.

[2] Zelenetz AD, Gordon LI, Abramson JS (2023). NCCN Guidelines® insights: B-cell lymphomas, version 6.2023[J]. J Natl Compr Canc Netw.

[3] Friedberg JW (2023). Update on follicular lymphoma[J]. Hematol Oncol.

[4] Alaggio R, Amador C, Anagnostopoulos I (2023). Correction: “The 5th edition of The World Health Organization Classification of Haematolymphoid Tumours: Lymphoid Neoplasms” Leukemia. 2022 Jul;36(7):1720-1748[J]. Leukemia.

[5] Cheson BD, Fisher RI, Barrington SF (2014). Recommendations for initial evaluation, staging, and response assessment of Hodgkin and non-Hodgkin lymphoma: the Lugano classification[J]. J Clin Oncol.

[6] Casulo C, Byrtek M, Dawson KL (2015). Early relapse of follicular lymphoma after rituximab plus cyclophosphamide, doxorubicin, vincristine, and prednisone defines patients at high risk for death: an analysis from the national lymphocare study[J]. J Clin Oncol.

[7] Jacobsen E (2022). Follicular lymphoma: 2023 update on diagnosis and management[J]. Am J Hematol.

[8] Khanlari M, Chapman JR (2022). Follicular lymphoma: updates for pathologists[J]. J Pathol Transl Med.

[9] Bachy E, Maurer MJ, Habermann TM (2018). A simplified scoring system in de novo follicular lymphoma treated initially with immunochemotherapy[J]. Blood.

[10] Luminari S, Ferrari A, Manni M (2018). Long-term results of the FOLL05 trial comparing R-CVP versus R-CHOP versus R-FM for the initial treatment of patients with advanced-stage symptomatic follicular lymphoma[J]. J Clin Oncol.

[11] Salles G, Seymour JF, Offner F (2011). Rituximab maintenance for 2 years in patients with high tumour burden follicular lymphoma responding to rituximab plus chemotherapy (PRIMA): a phase 3, randomised controlled trial[J]. Lancet.

[12] Sarkozy C, Baseggio L, Feugier P (2014). Peripheral blood involvement in patients with follicular lymphoma: a rare disease manifestation associated with poor prognosis[J]. Br J Haematol.

[13] Hu J, Gao F, Zhao J (2023). The prognostic index PRIMA-PI combined with Ki67 as a better predictor of progression of disease within 24 months in follicular lymphoma[J]. Front Oncol.

[14] Rummel MJ, Niederle N, Maschmeyer G (2013). Bendamustine plus rituximab versus CHOP plus rituximab as first-line treatment for patients with indolent and mantle-cell lymphomas: an open-label, multicentre, randomised, phase 3 non-inferiority trial[J]. Lancet.

[15] Flinn IW, van der Jagt R, Kahl BS (2014). Randomized trial of bendamustine-rituximab or R-CHOP/R-CVP in first-line treatment of indolent NHL or MCL: the BRIGHT study[J]. Blood.

[16] Margiotta-Casaluci G, Bigliardi S, Cocito F (2023). Comparison of first-line treatment with bendamustine plus rituximab versus R-CHOP for patients with follicular lymphoma grade 3A: results of a retrospective study from the Fondazione Italiana Linfomi[J]. Front Oncol.

[17] Mondello P, Steiner N, Willenbacher W (2018). Bendamustine plus rituximab versus R-CHOP as first-line treatment for patients with follicular lymphoma grade 3A: evidence from a multicenter, retrospective study[J]. Oncologist.

[18] Strati P, Ahmed MA, Fowler NH (2020). Pre-treatment maximum standardized uptake value predicts outcome after frontline therapy in patients with advanced stage follicular lymphoma[J]. Haematologica.

[19] Tilly H, Morschhauser F, Casasnovas O (2018). Lenalidomide in combination with R-CHOP (R2-CHOP) as first-line treatment of patients with high tumour burden follicular lymphoma: a single-arm, open-label, phase 2 study[J]. Lancet Haematol.

[20] Lyu R, Wang T, Zou D (2020). Long-term remissions of young patients with high-risk follicular lymphoma after first-line autologous stem cell transplantation: Three case reports[J]. Medicine (Baltimore).

